# Megalencephaly Polymicrogyria Polydactyly Hydrocephalus (MPPH): A Case Report and Review of Literature

**DOI:** 10.7759/cureus.16132

**Published:** 2021-07-03

**Authors:** Juan Fernando Ortiz, Samir Ruxmohan, Mahika Khurana, Jessica Hidalgo, Ivan Mateo Alzamora, Amrapali Patel

**Affiliations:** 1 Neurology, Universidad San Francisco de Quito, Quito, ECU; 2 Neurology, Larkin Community Hospital, Miami, USA; 3 Public Health, University of California Berkeley, Berkeley, USA; 4 Internal Medicine, San Francisco de Quito University, Quito, ECU; 5 Medicine, San Francisco University of Quito, Quito, ECU; 6 Public Health, George Washington University, Washington, USA

**Keywords:** polygiria, megalencephaly, polymicrogyria, hydrocephalo, -polycatylyia

## Abstract

Megacephaly polymicrogyria, polydactyly, hydrocephalus (MPPH) is an extremely rare condition caused by a defect in the AKT3, CCND2, or PIKR2 genes. Although the prevalence of the syndrome is very low, there is a significant clinical and radiological variation in the syndrome. We present a case with MPPH admitted to the hospital due to an increase in seizure frequency. The patient had a history of cerebral palsy, global developmental delay, spasticity, and hypoglycemic episodes. MRI findings revealed ventriculomegaly, polymicrogyria, abnormal encephalon, and pachygyria. The addition of clobazam and alprazolam diminished the seizures' frequency and the patient's spasticity, respectively. To highlight the clinical and radiological variation of the syndrome, we review cases of MPPH with clinical and radiological variants. Pachygyria and cerebral palsy are new associations not previously described before in MPPH. Pachygyria and cerebral palsy could be worsening the seizures and the global delay in this patient. Hypoglycemic episodes are probably related to the AKT3 gene, promoting more glucose consumption. Spasticity is most probably related to an upper motor sign due to the patient's cerebral palsy. This case highlights the clinical and radiological variation of the syndrome. More cases of MPPH need to be described to consolidate the knowledge and have a better understanding of the clinical and radiological variation of the syndrome.

## Introduction

Megalencephaly-polymicrogyria-polydactyly-hydrocephalus (MPPH) syndrome is a rare autosomal dominant syndrome that presents with multiple clinical and radiological features. The main features of the syndrome are perisylvian polymicrogyria, megalencephaly, postaxial polydactyly, and hydrocephalus [1-2]. At birth, the frontal occipital circumference ranges from normal to 6 standard deviations (SD) above the mean. In older individuals, the range is from 3 to 10 SD above the mean [1,2].

There are two types of pathologic variants seen in the occurring mutations; germline and somatic, with most having a germline pathogenic variant in one of the three heterozygous genes: AKT3, CCND2, or PIK3R2 [2,3]. The same genes cause Megalencephaly-capillary malformation (MCAP), a condition with similar clinical features with that of MPPH. Usually these two conditions overlap [3]. The main difference between these two disorders is hemihypertrophy and the presence of vascular malformations in MCAP [3].

The RAC-alpha serine/threonine-protein kinase (AKT) gene is located on chromosome 1 and encodes for a serine/threonine kinase, which it is mainly expressed in the central nervous system [4]. AKT is part of a PI3K-AKT-mTOR pathway and is important in cell growth, metabolism, brain development, synaptic plasticity, neurodevelopment, and cancer formation [3,4]. Most cases occur due to a mutation in the PIKT-AKT pathway. However, a second gene, CCND2, has also been implicated in 14 cases to this date [3]. CCND2 encodes cyclin D2 which regulates the PI3K-AKT pathway [3].

The clinical diagnosis is established in individuals based on two main features: megalencephaly and polymicrogyria. Hydrocephalus and polydactyly are complementary features to the diagnosis. The molecular diagnosis is established in a proband with some of the suggestive clinical and imaging features, and with the identification of one of the three heterozygous genes: AKT3, CCND2, or PIK3R2. Failure to detect either a germline or somatic mosaic pathologic variant in one of these three genes does not exclude a clinical diagnosis of MPPH syndrome in individuals with the clinical and imaging features [3].

Until now, only 62 cases of MPPH have been described. Even though the syndrome is extremely rare, the clinical variation and the MRI findings are important [4,5]. We are presenting a case of MPPH with cerebral palsy and pachygyria. These findings have not been described before in other cases of MPPH.

## Case presentation

History of present illness

A three-year-old male with a history of global developmental delay, epilepsy since age 1, and MPPH diagnosed a year ago came to the emergency department with status epilepticus for 45 min.

In the Intensive care unit (ICU), he developed cardiopulmonary arrest due to cardiac arrhythmia. He was intubated and then sedated with clonidine. He was diagnosed with MPPH at year one with a genetic due to his unusual clinical features (megalencephaly, global developmental delay, polydactyly)

The neurology team was consulted for abnormal twitching of the arms. A subsequent EEG showed abnormal intermittent slowing and high amplitudes with increased epileptiform activity in the right hemisphere. 

The seizures began with foaming in his mouth, oral twitching, and upward rolling of his eyes. The frequency of attacks is once every two months. Usually, the episodes last for 10-15 minutes. In the postictal state, the child is typically drowsy and confused.The infant had been receiving levetiracetam for seizure control.

In the past, the patient had two hypoglycemic episodes with glucose levels around 50mg/dL without any precipitating factors due to which the mother had to observe the child's diet. Additionally, the patient had cerebral palsy caused by an ischemic injury during birth due to umbilical cord-related asphyxia.

Past medical history

Prenatal History

No abnormalities were seen in the prenatal history.

Neonatal History

He was born at 38 weeks by cesarean section due to umbilical cord strangulation around his neck, which caused hypoxic injuries leading to cerebral palsy.

Seizure History 

At year one, an EEG showed abnormal spikes and waves. However, no seizures were seen. Three months later, he developed eye twitching due to which the patient was put on levetiracetam. A year ago, he started developing partial seizures with secondary generalization, with a frequency of once every two months.

Surgical History

Additionally, he has feeding and swallowing difficulties. He was also diagnosed with gastroesophageal reflux disease (GERD) for which he had to undergo Nissen fundoplication and feeding tube placement by gastrostomy.

Genetic Test

A genetic test showed that the patient had a pathogenic AKT3 mutation which supported the clinical and radiological findings in the patient.

Physical exam

Upon physical examination, the patient weighed 14.4 kg, was afebrile, awake, and obnubilate. Macrocephaly was easily noticeable (+3,5 SD), and there were some dysmorphic facies (small philtrum, small maxillar bones). He also had contractures in the upper extremities and increased tone, suggesting upper motor neuron disease. The rest of the physical exam was normal.

Neurological exam

The patient was lightly sedated but awake with eyes open spontaneously. There was not consistent visual attentiveness appreciated. He appeared to localize to sound but was unable to follow commands. There were limited spontaneous movements with diffusely increased extremity tone and developing contractures. He had decreased bulk for age. Regarding communication, he cried when angry, but the intensity of the sound is weak.

The child responded to tactile stimuli, and showed slight withdrawal to tactile and deep nailbed pressure in four extremities. On cranial nerve examination, II-XII were intact except for slightly deconjugate gaze. The reflex was diminished in the lower extremity; the Babinski sign was present.

Regarding milestone development on gross motor examination, he had difficulty walking and sitting; fine motor delay was also observed. He was unable to feed himself and could not transfer objects from one hand to another. When evaluating language, the child makes grunting sounds. Lastly, the child had poor social skills. He cried when he was hungry or wanted to go to the bathroom. He also made eye contact.

Diagnostic Assessment

Laboratory findings were as follows: White blood cell (WBC) count was 16.2 x 10^9^/L, hemoglobin (Hgb) was 8.1 g/dl, hematocrit (Hct) was 26.2%, platelet count was (Plt) 413 x 10^9^/L, Na was 135 mEq/L, K was 4.3 mEq/L, CO_2_ was 24.0 mEq/L, Cl was 105 mEq/L, and Cr was 0.20 mg/dL.

**Figure 1:**
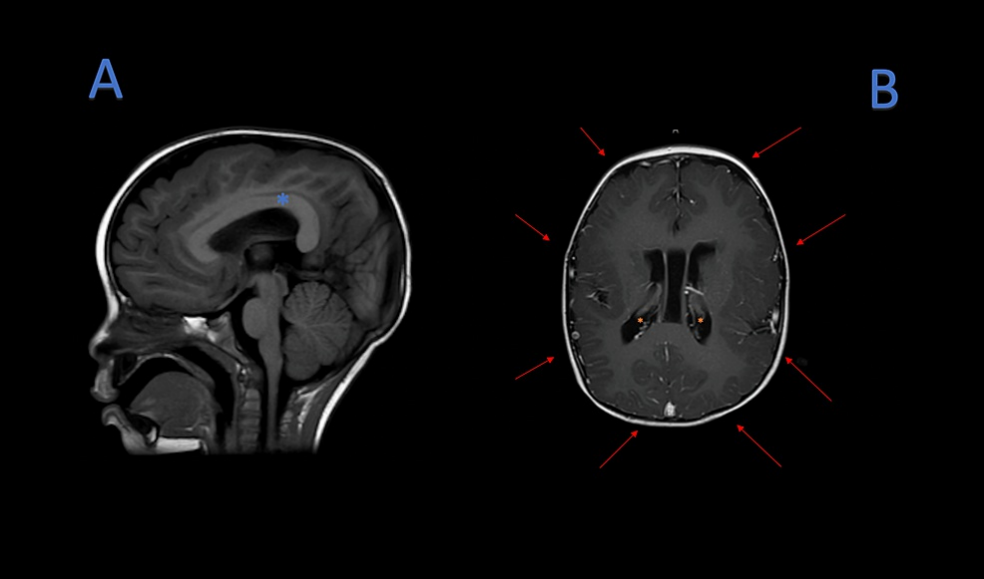
(A) MRI T1 sequence, shows thickening of the corpus callosum (blue asterix); (B) MRI T1 sequence, shows pachygyriam (red arrows), subependymal neuronal heterotopias (orange asterix). MRI: magnetic resonance imaging

Magnetic resonance imaging (MRI) of the brain showed abnormal morphology of the brain parenchyma with extensive polymicrogyria, subependymal gray matter heterotopia, and thickening of the corpus callosum, and pachygyria. Figure 1 highlights the subependymal gray matter heterotopia and thickening of the corpus.

**Figure 2:**
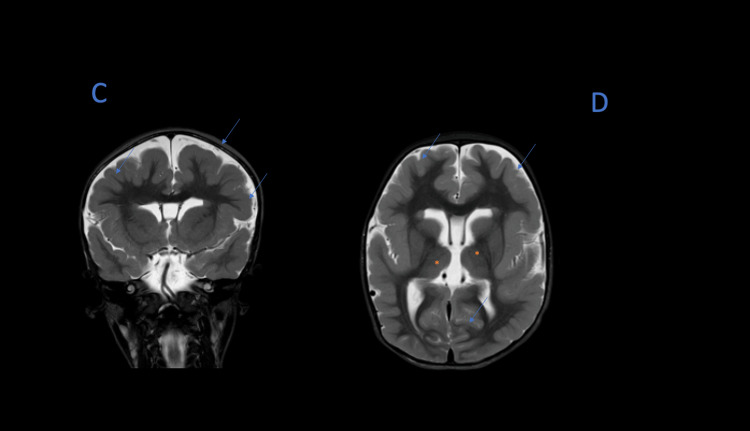
(C) MRI, T2 sequence, coronal view: Blue arrows show the polymicrogyria. (D) MRI, T1 sequence, sagital view: Blue arrows show the polymicrogyria and the orange asterix show the subependymal gray matter heterotopia MRI: magnetic resonance imaging

Figure 2 highlights the polymicrogyria, and the subependymal gray matter heterotopia.

**Figure 3:**
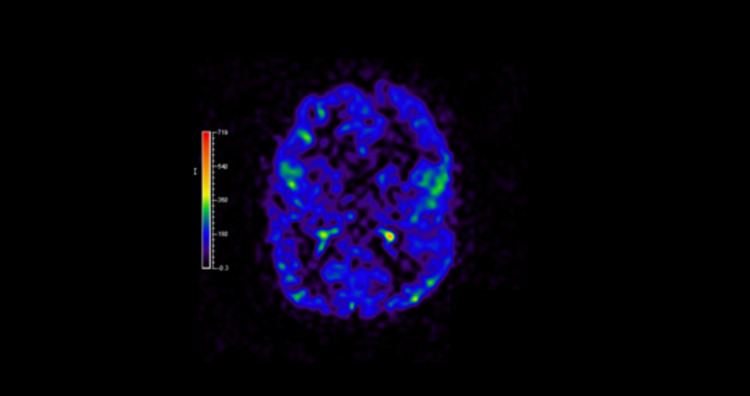
CT perfusion sequence. The scale represent areas with highest metabolism (red) and zones with lower metabolism (purple-black)

Figure 3 is a perfusion/restriction MRI that show the areas of metabolism of the brain in the infant.

A 24 hour electroencephalogram (EEG) showed multiple discharges with medium to high amplitude. The onset of the seizures was multifocal, especially in the left anterior temporal region and right-front central areas. These findings are suggestive of areas of cortical dysfunction leading to epileptogenic areas and correlates with the computer tomography (CT) perfusion sequence.

Impression, treatment, and follow up

This is a 3-year-old male with MPPH, cerebral palsy, with increase in seizure frequency. The cerebral palsy seemed to be contributing to the seizure problem, which was seen in the perfusion CT which showed many low metabolism areas. The patient also has interesting MRI findings besides the polymicrogyria. The patient has pachygyria and subependymal gray matter heterotopia, which could be worsening his condition as well. The team decided to add clobazam to the treatment regimen. The patient also had spasticity, for which he was put on diazepam. The diazepam improved spasticity. The patient was discharged after three weeks of hospitalization. During a phone call follow-up, the mother reported no seizures in the last month. The patient's prognosis is unclear due to the low number of cases of MPPH and his cerebral palsy.

## Discussion

Components of the syndrome

Megalencephaly is defined as an oversized and overweight brain that exceeds at least two standards deviations according to the patient's age and gender. Megalenchephaly needs to be differentiated from macrocephaly, which has a frontal-occipital diameter above two standards deviations [6]. Megalenchephaly can be classified as anatomic and metabolic megalencephaly. In metabolic megalencephaly, there is an accumulation of different metabolic components that increase the size of the brain. In contrast, in anatomic megalencephaly, a genetic component causes the clinical finding [6].

Polymicrogyria is defined by having multiple and small gyria. The pathogenesis is poorly understood, but it appears to be due to a problem in the development and migration of the neuroblast or glial cells. Cortical neurons usually migrate away from the origin in a radial and tangential fashion [7]. This migration seems to be disturbed in polymicrogyria. Polymicrogyria can be classified as local or diffuse and as unilateral or bilateral. There is also a subclassification depending on the region [7].

Polydactyly consists of “poly” meaning many and “dactily” meaning digits [8]. Polydactyly consists of having an extra finger or toe. This condition has been associated with 310 entities and 99 syndromes to date [9].

Hydrocephalus is defined as an accumulation of CSF inside the ventricles. This accumulation of fluid causes clinical symptoms in the patient [10] and can be caused by obstruction of the normal CFS pathways, problems with reabsorption of the fluid, or overproduction. As seen in our case, congenital hydrocephalus was caused mainly by obstruction. This type of hydrocephalus is commonly referred to as non-communicating hydrocephalus [10]. Besides obstruction, infections, prenatal hemorrhage, and more rarely, tumors can cause congenital hydrocephalus [10].

Table 2 shows conditions associated with megacephaly, polymicrogyria, and congenital hydrocephalus. Polydactyly is related to numerous conditions, so we decided to exclude polydactyly from the table [1-5].

**Table 1:** Clinical features and related disorders

Clinical Feature	Associated diseases or syndromes.
Anatomic Megaencephaly	Dwarfism, Sotos syndrome, Prezel syndrome.
Metabolic Megaencephaly	Canavan disease, Alexander disease, Tay Sachs, Sandoffs disease, Megalencephalic Leukoencephalopathy
Polymicrogyria	Walker Walberg syndrome, Aicardi syndrome, Zellweger syndrome, Beckwith-Wiedemann and Simpson-Golabi-Behmel syndromes, Sotos and Weaber (chromosome 5).
Congenital Hydrocephalus	Arnold Chiari type 1, Arnold Chiari type 2, agenesis of foramen of Monro, Dandy Walker malformation, Bickers-Adams syndrome.

Clinical variation of the syndrome

In table 3 we present some cases which highlight the clinical variation of the syndrome [3,4,11-15].

**Table 2:** Clinical variation of the syndrome

Author, Year.	Clinical Features	Radiological Features.	Atypical features and clinical variation in the cases
Sameshina et al, 2020 [3]	Male with no prenatal findings. Birth weight was 4.102g (+3,8 SD), OFC (+ 4.2 SD) Diagnosed at 1 year. Develop epilepsy at year one	Enlarged ventricles, polymicrogyria.	He presented with coartation of the Aorta at birth. He had a heterozygous missense variant in CCND2 gene. No PI3K-AKT mutation.
Cappuccio et al, 2019 [12]	Male, prenatally ultrasound revealed enlarged ventricles and polyhydramnios. OFC (+3SD). He develops infantile spasm at 7 months. Diagnosed at one year.	Dysmorphic midbrain reduced volume of white matter, hippocampal hypoplasia, polymicrogyria, thinned corpus collosum, and hypoplasia of the pons.	Mutation in CCND2 gene. Patent foramen ovale and ductus arteriosus that eventually close after 1 year. Bilateral cryptorchidism.
Demir et al, 2015 [13]	Female, prenatal ultrasound revealed ventriculomegaly. Birth weight was 3900g and OFC (+4SD), During the neonatal period he was in the ICU due to respiratory distress. He developed seizures since the first month of life. At day 46 he died of sepsis and DIC.	Ventriculomegaly. United ventricle, occipital hydrocele, polymicrogyria, and thin cerebral parenchymal structures.	The patient presented with occipital encephalocele, cleft palate. He also had multiple polyps in the tongue. No genetic testing was conducted.
Osterling et al, 2011 [11]	No prenatal findings, OFC (+4SD). He de developed infantile spasms at 11 months of age. General developmental delay.	Ventriculomegaly, polymicrogyria, extensive cortical dysplasia, polymicrogyria in frontal, temporal, partial and opercular regions. Cavum septum pellucidum, thinning of the corpus collosum	Chiari 1 malformation
	Prenatally it was seen macrocranea with normal size ventricles. OFC (+5SD). Abnormal EEG, but no diagnosis of epilepsy.	Ventriculomegaly, perysilvian cortical dysplasia in the frontal, parietal, and temporal lobes. The patient also had polymicrogyria	Medulloblastoma in the posterior fossa.
Pisano et al, 2008 [14]	No prenatal abnormalities. OFC (+3SD). Developmental delay. A ventriculoperitoneal shunt was placed at eight months. EEG showed normal discharges, but he never presented seizures.	Enlargement of the third and lateral ventricles. Cavum septum pellucidum, mega cerebellum, bilateral abnormal white matter intensities, and diffuse polymicrogyria.	Syndactyly is the hands and feet.
Hadzipasic et al, 2021 [15]	Data could be extracted	Hydrocephalus, Polymicrogyria, Megaencephaly.	Medulloblastoma in the posterior fossa
Szalai et al, 2020 [4]	No prenatal complications. Macrocephaly at birth. He developed seizures at age 8. He also has motor and speech developmental delay. heterozygous missense variant in the AKT3 gene	Chiari malformation, no hydrocephalus, periventricular leukomalacia, perysylvian polymicrogyria.	Chiari type I malformation.
	No prenatal abnormalities Required oxygen at birth. OCP (+97 percentile) heterozygous missense variant in the AKT3 gene.	Lateral ventricle asymmetry, periventricular heteropias, perysylvian polymicrogyria.	Partial syndactyly in the 4^th^ and 5^th^ finger.

There was substantial variation in the MPPH cases, clinically and radiologically. However, there were some repeated associations in these cases. Medulloblastoma was reported in two cases [11,15]. At the same time, Chiari Type 1 malformation was also seen in two cases [4,11]. Extra-neurological manifestations such as syndactyly were seen in two cases [4,14]. Cardiological manifestations were seen in two cases as well [12,13].

There was clinical variation among siblings: in the cases reported by Osterling et al., the first sibling presented epilepsy and Chiari 1 malformation, while the second one did not present seizures and had an associate medulloblastoma in the posterior fossa [11]. MRI findings were also different between the two siblings [11].

The two cases reported by Szalai et al. showed the phenotypical variation of two siblings with the same mutation. The abnormally in the first case was Chiari type 1 and in the second sibling syndactyly in the fourth and fifth finger [4].

Clinical variation of the case

The clinical presentation in MPPH varies even though there are only 62 cases described to date. Expected findings in the case were feeding difficulties, seizures, megalencephaly, polymicrogyria, abnormal encephalon, and global developmental delay.

Unexpected findings in the case were the hypoglycemic episodes and the co-existence of cerebral palsy. MRI features not described before in MPHH in our patient were: pachygyria and subependymal neuronal heterotopias.

*Epilepsy and Developmental Dela*y

These two clinical features are common as the disease progresses, so this finding was not unusual in our patient [3]. Epilepsy is seen in 50% of cases with MPPH, while the developmental delay is almost universal in all cases.

Hypoglycemic Episodes

This characteristic has been described previously. Nellist et al. described a case of an 11 month-year-old with a head circumference of 7 SD above the mean [2]. At six months, the infant developed infantile spasms resistant to multiple therapies with vigabatrin, adrenocorticotropic hormone (ACTH), levetiracetam, and clobazam [2]. The child also had persistent episodes of hypoglycemia. A novel, de novo AKT3 missense variant (exon 5; c.548T>A, p.(V183D)) was identified and shown to activate AKT3 by in vitro functional testing which led to increased glucose utilization due to activation and caused the sustained hypoglycemia [2].

Cerebral Palsy

Cerebral palsy is a nonprogressive disorder which occurs in the developing fetal or infant brain. Motor disorders are the most common manifestations but can also be accompanied by disturbances in sensation, cognition, and communication [16]. While the AKT3 mutation does not cause cerebral palsy, it is relevant in this case because cerebral palsy increased the risk of epilepsy [16]. It is important in this case for the prognosis of the seizures in this patient. The coexistence of both conditions has not been described before. Certainly, cerebral palsy, in this case, was caused by birth asphyxia.

Pachygyria

The MRI findings like thick corpus callosum, polymicrogyria, abnormal encephalon, and gray matter heterotopia have been described before in MPPH. However, pachygyria has not been described in MPPH. Pachygyria is a congenital malformation that causes thick convolutions in the cerebral hemisphere [17]. Pachygiria is associated with causing seizures, poor motor control, global developmental delay, and feeding/swallowing difficulties [17]. Pachygyria contributed to the seizures in this patient and may also be a contributing factor in the feeding difficulties of the patient.

## Conclusions

MPPH is an extremely rare condition. However, the syndrome presents important clinical and radiological variations. Chiari type 1, medulloblastoma were some of the neurological findings found in our review. Syndactyly and heart abnormalities were also part of the clinical variability of the syndrome. The MRI findings like thick corpus callosum, polymicrogyria, abnormal encephalon, and gray matter heterotopia were expected MRI findings in the case as compared to others. Epilepsy and global developmental delay are also common clinical features. However, the co-existence of cerebral palsy and pachygiria were unique findings, making this case novel and unique. Cerebral palsy and pachygyria seem to be contributing factors for the patient's epilepsy. More reports of MPPH need to be further documented for a clear picture of the clinical variability of the syndrome.
